# Extending screening intervals for women at low risk of breast cancer: do they find it acceptable?

**DOI:** 10.1186/s12885-021-08347-w

**Published:** 2021-05-29

**Authors:** Lorna McWilliams, Victoria G. Woof, Louise S. Donnelly, Anthony Howell, D. Gareth Evans, David P. French

**Affiliations:** 1 Manchester Centre for Health Psychology, Division of Psychology & Mental Health, School of Health Sciences, Faculty of Biology, Medicine and Health, University of Manchester, MAHSC, Oxford Road, Manchester, M13 9PL UK; 2 Nightingale Breast Screening Centre & Prevent Breast Cancer Research Unit, Manchester University NHS Foundation Trust (MFT), Southmoor Road, Manchester, Wythenshawe M23 9LT UK; 3 NIHR Greater Manchester Patient Safety Translational Research Centre, Centre for Mental Health and Safety, School of Health Sciences, Faculty of Biology, Medicine and Health, University of Manchester, MAHSC, Oxford Road, Manchester, M13 9PL UK; 4 Department of Genomic Medicine, Division of Evolution and Genomic Science, MAHSC, University of Manchester, Manchester University NHS Foundation Trust, Oxford Road, Manchester, M13 9WL UK

**Keywords:** Breast cancer, Risk assessment, Risk-stratification, Acceptability, Screening, Mammography, Risk communication

## Abstract

**Background:**

Trials of risk estimation in breast cancer screening programmes, in order to identify women at higher risk and offer extra screening/preventive measures, are ongoing. It may also be feasible to introduce less frequent screening for women at low-risk of breast cancer. This study aimed to establish views of women at low-risk of breast cancer regarding the acceptability of extending breast screening intervals for low-risk women beyond 3 y.

**Methods:**

Semi-structured interviews were used to explore views of low-risk women, where “low-risk” was defined as less than 2% estimated 10-year risk of breast cancer aged > 46 years. Low-risk women were identified via the BC-Predict study, where following routine screening, women were given their 10-year risk of breast cancer by letter, along with additional information explaining breast cancer risk factors. To gain diversity of views, purposive sampling by ethnicity and socioeconomic background was used to recruit women. Data were analysed using thematic analysis.

**Results:**

Twenty-three women participated in individual interviews. Three themes are reported: (1) *A good opportunity to receive risk estimation*, where women found it worthwhile to receive a low-risk result although some were surprised if expecting a higher risk result; (2) *Multi-faceted acceptability of extended screening intervals*, with reactions to less frequent screening dependent on whether women were confident in being low-risk status and current safety evidence, (3) *Passive approval* versus *informed choice*, highlighting that women found it difficult to consider choosing less frequent screening without professionals’ recommendations, as they generally viewed attending breast screening as positive.

**Conclusions:**

Risk assessment and receiving a low-risk of breast cancer is acceptable although, further research is required with more diverse samples of women. Any recommendation of less frequent screening in this risk group should be evidence-based in order to be acceptable. Communication needs to be carefully developed, with a focus on ensuring informed choice, prior to trialling any extended screening recommendations in future studies.

**Supplementary Information:**

The online version contains supplementary material available at 10.1186/s12885-021-08347-w.

## Background

The UK NHS Breast Screening Programme (NHSBSP) invites women 3-yearly from age 50–70 years [1]. This has not been without controversy, centring on whether such programmes reduce overall mortality or burden of treatment from early detection [2, 3] and whether this outweighs harms. Specifically, screening programmes may detect cancers that would have never become symptomatic to require treatment i.e., overdiagnosis/overtreatment [4] or lead to false positive screening test results [5], with lasting psychological impact [6]. Given this controversy, efforts have been made to clearly communicate about these potential harms and benefits of screening [7]. Despite this, a recent review concluded that women tend to view the benefits of breast screening as outweighing the harms, and many misunderstand the concept of overdiagnosis [8].

There are specific UK national guidelines to offer additional screening to women at higher risk of breast cancer [9]. However, only around 1 in 6 women at moderate/high-risk of breast cancer have a family history (FH) of the disease consistent with NICE FH criteria, identified generally when women present to primary care and are referred to family history, risk and prevention clinics [10]. The use of risk estimation models (for example, Tyrer-Cuzick) allows 5-year, 10-year or lifetime breast cancer risk to be calculated in a timely manner, and thereby identify these moderate/high-risk women [11, 12]. Given this, interventions to systematically identify women at higher risk of breast cancer as part of routine breast screening, are currently being evaluated [13, 14]. For example, the BC-Predict intervention provides feedback to women according to four risk categories: below average (or low), average, above average (or moderate) and high.

Although much of the impetus for risk-stratified screening comes from the potential to identify high risk women and offer them prevention and early detection options, discussion about the implementation of a risk-stratified breast cancer screening programme (BCSP) has led to consideration of whether it is safe to reduce screening for women at low-risk. Not only are such women by definition less likely to develop cancer, they are also less likely to develop faster growing, high-grade tumours [15]. Ensuring women understand the harms and benefits of a risk-stratified BCSP, where screening invite frequency could be reduced for those low-risk, is arguably even more important should it be introduced more widely. UK professionals involved in national screening programme policy decision-making and breast screening programme service delivery reported acceptability of extended breast screening intervals for low-risk women in principle, although identified key implementation issues, including obtaining acceptability from women [16, 17].

Although previous surveys indicate women think risk-stratified breast screening is a good idea, the samples were more accepting of additional screening if high-risk than less frequent screening if low-risk [18, 19]. However, it was unclear why women were less willing to accept this. Although previous qualitative studies have discussed risk-stratified breast cancer screening with women of breast screening age [20–22], there appears to be no published research directly exploring the views of women with a low-risk estimate. This is notable, as if extending the screening interval is unacceptable to the affected population, it would be difficult for this change to be feasible. The present study therefore aimed to elicit the views of women at low-risk about receiving this information, and their acceptability of less frequent breast cancer screening invites.

## Materials and methods

### Design and participants

A cross-sectional, qualitative design using semi-structured interviews was employed. Women were identified from an on-going study investigating breast screening risk stratification, BC-Predict [14], and had consented to be approached about future research. In BC-Predict, women were invited to find out their 10-year risk of breast cancer as part of routine breast screening. Consenting BC-Predict participants complete a self-report risk estimation questionnaire (online or paper) and approximately 6-weeks later (if the participant has received a clear mammogram result), receive written feedback based on information they provided about known breast cancer risk factors and breast density calculated from their mammogram images.

### Procedure

Women were invited by post to participate in an interview between 23 and 246 days after having received their BC-Predict risk feedback. Only women with a 10-year risk estimate of < 2% were invited to participate i.e., low-risk. To recruit a diverse sample, up to two additional reminder letters were posted to women who self-reported Black and Minority Ethnic Refugee ethnicity or had an Index of Multiple Deprivation (IMD) [23] decile of below 5 (1 indicates the most deprived and 10, the least).

Interviews took place in participants’ homes, in a quiet room (university or hospital), or by telephone. All interviews were conducted by two female researchers with post-graduate experience conducting qualitative research. With consent, they were audio-recorded, transcribed verbatim and each woman assigned a pseudonym. Data collection continued until research team members (LM, VGW, DPF) agreed there was sufficient data to answer the research aim [24].

### Materials

To develop an interview topic guide, women approaching the age at which they would be invited by the NHSBSP reviewed a draft topic guide based on a review of relevant literature and, findings from previous interviews conducted with national screening figures [16] and screening professionals [17]. Feedback on wording and order of questions were incorporated and refined. The final version (Additional file 1) focused on asking women about their experience participating in BC-Predict, receiving a low-risk breast cancer estimate, views about reducing the screening frequency for low-risk women (including decision-making, communication and information needs) and, understanding of the harms and benefits of breast screening. The guide was used flexibly in each interview to follow up ideas and thoughts introduced by participants.

### Analysis

Interview data was analysed using reflexive thematic analysis from a critical realist perspective, based on the reality of the data and acknowledging the researchers’ role in analysis [25]. Transcripts were read, re-read and audio-files listened to with initial notes captured, before manifest-level, inductive coding using Nvivo 11 software. Two transcripts were double-coded (LM, VGW) and any discrepancies discussed and resolved as the iterative coding framework developed (LM). Coding was used to generate candidate themes compared across the data set and continually discussed (LM, VGW, DPF) before refinement. The final thematic structure was agreed by the entire study team as representing participants’ views.

## Results

### Sample

Of 83 women invited, 26 indicated interest and 23 took part. Interviews lasted 33 to 107 min (median 68 min), with three conducted by telephone and 20 face-to-face. Fourteen women took part following their first BCSP invite. For additional sample details, see Table 1.

**Table 1:** Sample characteristics

Characteristic	Number of women (*N*=)
*Age*	
46–54 years	16
55–64 years	3
65–74 years	4
*Ethnicity*	
White British	19
Asian or Asian British: Indian	1
White European	1
Black or Black British: African	1
Mixed: White & Black African	1
*Education*	
A levels or equivalent	2
Diploma e.g. nursing, teaching	3
Degree	10
Postgraduate certificate/diploma	2
Postgraduate degree	5
*Index of Multiple Deprivation Decile*	
1 (most deprived)	1
2	2
3	4
4	1
5	7
6	0
7	0
8	6
9	1
10 (least deprived)	1

Three themes were produced: (1) A good opportunity to receive risk estimation, (2) Multi-faceted acceptability of extended screening intervals and (3) Passive acceptance versus informed choice. Quotes are presented with a pseudonym followed by each participant’s age bracket.

### Theme 1: a good opportunity to receive risk estimation

The sample revealed how they felt about being at low-risk of breast cancer as part of breast screening, were able to describe what it meant personally and, how this impacted on their views about extended screening intervals.

#### Reactions to personal risk estimates: two sides of the same coin

All women reflected on receiving a low breast cancer risk estimate positively, although many could not remember exact contents of feedback letters. Understanding the different contributions of risk factors largely underpinned how women interpreted the feedback and, whether they expected their result. Where women were surprised, they had expected their family history or other risk factors (e.g., not having children) to contribute to receiving a higher estimate and questioned the accuracy of risk assessment. Others wondered how the different risk factors are weighted and on two occasions, women described providing risk information as difficult.

*…what’s not clear from the information, is how much that prediction is based on the actual mammogram results and how much is based on what you completed in your questionnaire Erinma;45-54*

Overall, women explained that their results provided a sense of relief and for many, ‘*one less thing’* to worry about. In one account, a woman described her risk as helpful when deciding about medication for menopausal symptoms.

*I kind of felt more reassured about going onto [Hormone replacement therapy] HRT because of the prediction said low [...] going on HRT may not be that bad because although it may be only an increase of one in a 100 people, women, who get breast cancer from being on HRT, I’m already at a low level. Shelagh;45-54*

#### Minimising complacency

Due mainly to receiving validation of how they already felt, some women were somewhat dismissive re-counting the experience as not having much impact *…perhaps you don’t take it in so much because it’s not a direct immediate threat to your own health (Emma;45–54).* Despite this, many recognised that receiving low-risk breast cancer estimates does not mean that they will never be diagnosed and although helpful information to receive, expressed realistic views that they could still be diagnosed with breast cancer. Subsequently, most voiced intentions to maintain healthy lifestyles, attend future screening and remain breast aware.

*It means that, sort of, lifestyle, things that have…you know, things that have happened in the past genetically, I’m probably at a lower risk of getting breast cancer, but anyone can get it still. It could happen at any time so it’s not that I’m never going to get it, it’s just that I’m at, sort of, the low risk end of the scale of likely to get it.* Annie;45-54

#### Cautiously positive about extended intervals

Initial reactions to the idea of less frequent screening were positive, or neutral, across most of the sample. Women who did not expect to receive a low-risk estimate, and who wanted to know more about the stability of their risk, were more apprehensive when discussing longer screening intervals. Several questioned whether longer intervals are being considered for cost cutting reasons, rather than safety. This concern led to some expecting a backlash from women feeling entitled to remain on 3-yearly intervals.

*Actually, the truth of the matter is I think I’d rather feel a bit miffed about it […] my immediate reaction would be ‘it’s a money saving’ […] Maybe I’m being cynical, but I take it as being a money saving device.* Violet;65-74

In contrast, assuming that longer screening intervals would allow those at higher risk of breast cancer to be offered more screening, others viewed the proposal altruistically, as a better use of national funding. Regardless of viewpoints, women wanted to know more about why less frequent screening for low-risk should be introduced.

*…it’s just me using my brain isn’t it? They’re at greater risk; give them the chance to have it caught early and then if somebody doesn’t have to go through full blown chemotherapy and maybe can just potentially have radio [therapy].* Enriquetta;45-54

### Theme 2: multi-faceted acceptability of extended screening intervals

Women considered what breast screening could look like if low-risk women were offered less frequent screening and, thoughts were generally based on their opinions about current breast screening. This was challenging for women to discuss without having previously thought much about how screening programmes are generally organised.

#### Screening means security

Despite being largely accepting of the possibility of less frequent breast screening, women viewed the current 3-yearly programme favourably overall. Some women acknowledged that interval cancers and false positives already exist within 3-yearly screening however, concerns about having fewer mammograms related to a loss of safety that breast cancer will be detected quickly. Women often cited present life circumstances, or knowing individuals who have experienced cancer, as reasons to continue with 3-yearly screening invites.

*…at my age now I’d feel a duty towards my family, my children, that I should go through treatment and that’s how I feel […] really catching any cancer early would be important to me, as most people, I guess, but especially the younger you are. Maxine;45-54*

Therefore, in spite of being given information that those at low-risk could be less likely to be diagnosed with ‘aggressive’ breast cancer, women were worried that longer intervals would result in later stage cancer diagnoses. Communication about longer screening intervals, emphasising the importance of self-examination with a *‘I am low risk, there’s no history in the family, but it doesn’t mean to say I’ll never get it’ (Elizabeth;55–64)* approach, was expected to minimise interpreting the change as license to ignore breast health. On two occasions, women highlighted the need for a clear rationale from experts to minimise contradicting the early detection agenda initiatives aimed at improving screening uptake.

*…you are very much encouraged to go for any screening that’s available and I think knowing what kind of breast cancer can involve in treatment and things like that.* Lydia;45-54

Improvements in life expectancy led some to state that the upper age limit (70 or 73 years) is no longer fit for purpose and, introducing low-risk screening provides opportunity for reviewing the entire programme.

*…people are living longer and it used to be that people died between 50 and 70…shift everybody along a bit.* Eleanor;65-74

#### A reasonable screening length

All women discussed what screening interval length could be offered, with quite varied opinions. Everyone felt there should be *some* screening once low-risk has been established. However, many were unsure what the current interval or invite age range is and how these were decided. It was quite difficult for women to elicit a specific interval although expected it to be underpinned by evidence.

*I would kind of assume that lots and lots of research and thinking had gone on behind all of that, it wouldn’t just be a magic figure plucked from thin air.* Ellen;45-54

When prompted by the interviewer, an interval of 5 years was viewed most positively although, many were unable to articulate why; 10 years seemed too long for most. Some women referenced age-adapted cervical screening intervals to explain their views and often highlighted they have not questioned the lengths in this programme. Others felt a change should not be too drastic as women are already experiencing health changes.

*I suppose it seems like a big gap to me, six years seems quite a lot […] I’m currently going through the menopause, and the amount of physical change that has happened over the last year is quite staggering.* Marie;45-54

A number of women wondered whether low-risk screening frequencies would be different depending on age and whether risk could change over time. Reasoning conflicted relating to perceptions that older women are at greater risk but, breast cancer diagnoses would have a greater negative impact on younger women. Given that risk assessments would identify higher risk women (to offer more frequent screening), two women suggested risk-stratified screening should start earlier than age 50-years.

*…instead of waiting until 50 you’re almost selling it as a, ‘we’re actually going to screen you early’ […]at 45 and then every five years after that, unless the risk changes* Victoria;45-54

### Theme 3: passive approval versus informed choice

When asked how they would decide whether to opt for longer screening intervals, women identified the limits of ensuring informed choice within screening as felt unaccustomed to deliberate about how much they interact with such programmes. This led women to expect that guidance from healthcare professionals in the field would be provided.

#### Individual decision-making is challenging

Deciding whether to accept longer screening intervals was often difficult for women to consider. Many women could not remember the harms (i.e., overdiagnosis and false positives) and benefits of breast screening provided with their invite and, most viewed false positives as good in order to rule out cancer.

*I mean I wasn’t aware that there was any deleterious effect from the actual screening process. So under those circumstances I think I’d probably prefer to carry on being screened.* Emmeline;65-74

Whether it is possible to implement a ‘strict’ longer screening interval, where women are not offered choice, influenced many women’s thoughts about longer intervals. Knowing they could easily re-access screening with suspected symptoms, and be taken seriously, were assumed by many:

*…if I was to feel something that I’m not used to within my body, just because my screening is in five years, I don’t have to wait for five years, I can go to the GP and say, 'look, this is not comfortable', the GP will refer […] It’s not, like, 'this is it, we’ve set it in stone' and it cannot be changed.* Clara;45-54

Regardless, when asked what decision they would make if it were a choice, several women initially ‘on the fence’ when first discussing longer intervals, reported that they would be *‘quite happy to have it every three years’* (Carol;45–54) for reassurance. In one account, a woman viewed it as unfair to not be allowed to make her own decision despite acknowledging that the cervical screening interval changes depending on age. However, others wanted to be told how frequently they should attend mammograms as would feel unable to decide on their own:

*I’m a natural born worrier, so I then think back to the research that says, 'well it might be more harmful to have them more frequently'. Then I’d be torn as to, 'oh what do I do, it could be more harmful to have them every three years?' […] Or, what if I’m the one that’s low risk and does get cancer? I’m the one that’s low risk and gets a false positive because I’ve been going every three years?* Lucy;45-54

#### Desire for guidance from professionals

Given the complexities women highlighted when considering how they would opt for extended screening intervals, provided a clear, evidence-based explanation was given, the majority described feeling accepting of the idea that less frequent screening for low-risk might be introduced.

*…if the NHS feel confident in giving that advice then I would feel confident in taking because you are a trusted supplier of messages.* Joanne;55-64

Without having been able to review evidence demonstrating the safety of less frequent screening, one woman explicitly disagreed with this.

*So if they automatically said every five years I’d probably be a bit annoyed, just because there is going to be a percentage of people who do get it. Not annoyed, but I’d be a bit worried. If I was given the choice and made my own decision based on more accurate details and facts and then that would be my decision, rather than every three years, then I’d be happier with that than just being told ‘you’re in that category, we’re going for every five years’*. Caroline;45-54

Women viewed the proposal as placing greater responsibility on women for their breast health therefore, a number identified a default longer interval with the ability to opt back in to 3-yearly intervals could improve acceptability; one woman suggested this could be temporary ‘…*maybe that for a period of transition might be the solution rather than having a big outcry of women being deprived mammograms (Jackie;45–54).* However several wondered if this would be possible financially.

*Is it a good use of resources? I mean if it turns out that a larger number of women want to stay on the three-year cycle and yet professionally it’s not considered necessary, it’s a tricky one. […] If it were very low numbers, it wouldn't matter too much. But it doesn't seem right to be offering some expensive procedures or whatever when they are not medically necessary.* Ethel;65-74

## Discussion

Overall, women felt generally positive about breast cancer risk being assessed as part of routine breast screening and receiving a low-risk estimate. Less frequent screening for women at low-risk appeared acceptable, as long as safety evidence is available and is not only due to saving money. Women did not believe screening should stop altogether for this group and indicated an interval length not too dissimilar to the current 3-yearly programme could be appropriate. It was difficult for most women to think about deciding to choose a longer interval; most wanted guidance from expert health professionals. Women appeared to base their views about the acceptability of less frequent screening on their experience of others having breast cancer, their understanding of the BCSP and how they currently interact with screening.

Women appreciated the chance to receive their 10-year risk of breast cancer and the majority were pleased with receiving a low-risk estimate. This is in line with findings of a previous quantitative study that found that women told they were at lower risk felt less cancer worry and general anxiety than other women [26]. However, some women were less convinced by their result had they expected to receive a higher risk or, were unsure they had been able to provide adequate information to allow accurate risk estimates to be produced. This generally underpinned how women reacted to the idea of having less frequent breast screening based on these estimates. Consistent with previous research focusing on extending screening for women at low-risk of breast cancer [16, 17] and studies exploring risk-stratified breast screening as a whole [21, 27], women sought evidence that demonstrated the safety, including whether estimates are accurate and stable over time. Although evidence illustrates the accuracy of 10-year breast cancer risk models for predicting cancer [28], how much these risk estimates change for individuals is poorly understood.

Although balanced information is likely to be included in invites to breast screening, women with positive attitudes towards cervical screening downplay the harms [29], so it is not surprising women in this study did so when almost the entire sample viewed breast screening positively. Similarly, it was to be expected that women hesitate to identify a longer screening interval length for those at low-risk as concepts such as overdiagnosis are largely difficult to comprehend [30]. In the present study, many questioned why the current interval is 3-years, suggesting a general passive acceptance of screening invites. Women found it difficult to consider making a decision about whether to have less frequent screening or, opt for the current 3-yearly interval, with some suggesting they would remain on 3-years just in case. This echoes previous research indicating that although risk-stratified breast screening was viewed positively by women who have not participated in breast cancer risk assessment, there was less enthusiasm for reduced screening if low-risk [18, 20]. Previous research has shown American and Australian women have suspicions that breast screening programme changes are underpinned by money-saving efforts [31, 32]. However, some considered less frequent screening altruistically where resources could be re-allocated to those in greater need, which may be based on the number of women who cited others affected by the disease.

### Strengths and limitations

The study explored views about risk-stratified breast screening from women who with a low 10-year breast cancer risk estimate; other research has focussed on women with average or above risk. Recruitment methods aimed to ensure a diverse sample from an ethnicity and socioeconomic perspective but, were limited by those eligible from the BC-Predict study. Over 80% of the eligible sample were of White British background, reflecting the current study sample however, the recruitment approaches used may not have been the optimal method of engaging with non-White groups of women. The median IMD decile (five) for the sample interviewed matched that of BC-Predict women invited to the study and, recruitment rates were slightly higher (29%) for women living in areas with an IMD ranging from 1 to 5 than those living in less deprived areas (24% for IMD range 6–10). Women who took part were, on the whole, highly educated and were willing to have their breast cancer risk assessed. It could be that this sample are particularly interested in breast cancer risk and could be more accepting of extended screening intervals however, the interview topic guide was open allowing positive and negative views to be expressed. Due to the lack of ethnic diversity within both the recruited sample and BC-Predict, it may be that non-White women have particular views about the acceptability of breast cancer risk assessment as well as less frequent screening for women at low-risk.

### Implications

Any risk-stratified screening approach where screening is reduced for low-risk women should clearly explain the rationale and supporting evidence given that having adequate knowledge partly underpins informed choice [33]. Some women were concerned that the proposal was financially-based. Despite reservations, the women appeared generally trusting that a publically funded programme would only recommend this change if appropriate to do so. Communication around such changes to breast screening, that could be considered de-implementation of a service, should however be carefully handled especially to minimise misunderstanding, as highlighted in interviews with UK screening figures and screening professionals [16, 17]. In addition, communication materials should be developed in consideration of the ethnic diversity of the population likely to be invited given that previous research identified that British-Pakistani women were enthusiastic about risk-stratified screening [34] although described barriers to attending the NHSBSP including lack of knowledge about breast screening [35].

Further research is required to assess how women might respond to a recommendation of extending BCSP screening intervals in receipt of a low 10-year breast cancer risk estimate. Views from women who do not currently adhere to screening recommendations i.e., invited to the BCSP but do not attend for a mammogram, should be obtained to determine whether risk-stratified screening may be more or less acceptable if a low-risk pathway is introduced. This is particularly important given the high educational attainment of the present sample as evidence suggests lower uptake and poorer understanding of risk is associated with socioeconomic status, such as qualifications [36]. Future research should be conducted with women from diverse backgrounds as although previous research highlighted that British-Pakistani women were interested in breast cancer risk assessment, including many where English was a second language, views may vary across other ethnic groups [34].

## Conclusions

The findings have demonstrated that it is acceptable to receive a low-risk breast cancer estimate as part of breast screening, from the perspective of a generally well educated, mainly White British sample. Several challenges were highlighted in ensuring informed choice should risk-stratified screening approaches to breast cancer screening introduce less frequent screening for women identified at low-risk of breast cancer.

## Supplementary Information

**Additional file 1.** Interview topic guide

## Data Availability

The datasets used and/or analysed during the current study available from the corresponding author, on reasonable request.

## Abbreviations

BCSP: Breast cancer screening programme
FH: Family history
GP: General practitioner
HRT: Hormone replacement therapy
IMD: Index of Multiple Deprivation
NHSBSP: NHS Breast Screening Programme
NICE: National Institute for Health and Care Excellence
UK: United Kingdom
