# Supplementary material for: Extending screening intervals for women at low risk of breast cancer: do they find it acceptable?
Source: BMC Cancer. 2021 May 29;21:637. doi: 10.1186/s12885-021-08347-w (PMC8164783; doi:10.1186/s12885-021-08347-w)
Supplement: Supplementary file 1 — Additional file 1. Interview topic guide [file 12885_2021_8347_MOESM1_ESM.doc]

**Title:** Extending screening intervals for women at low risk of breast cancer: do they find it acceptable?

**Lorna McWilliams^1*^, Victoria G Woof^1^, Louise S Donnelly^2,4^, Anthony Howell^2^, D Gareth Evans^2,3^, David P French^1^**

*****Corresponding author.

Email address: [lorna.mcwilliams@manchester.ac.uk](mailto:lorna.mcwilliams@manchester.ac.uk)

^1^Manchester Centre for Health Psychology, Division of Psychology & Mental Health, School of Health Sciences, Faculty of Biology, Medicine and Health, University of Manchester, MAHSC, Oxford Road, M13 9PL, Manchester, UK

^2^Nightingale Breast Screening Centre & Prevent Breast Cancer Research Unit, Manchester University NHS Foundation Trust (MFT), Southmoor Road, Wythenshawe, M23 9LT, Manchester, UK

^3^Department of Genomic Medicine, Division of Evolution and Genomic Science, MAHSC, University of Manchester, Manchester University NHS Foundation Trust, Oxford Road, M13 9WL, Manchester, UK

^4^ NIHR Greater Manchester Patient Safety Translational Research Centre, Centre for Mental Health and Safety, School of Health Sciences, Faculty of Biology, Medicine and Health, University of Manchester, MAHSC, Oxford Road, M13 9PL, Manchester, UK

**Additional File 1 Interview topic guide**

**Interview questions and discussion points**

**Experience of BC-Predict**

1. Can you tell me what you remember about taking part in the BC-Predict study (NB: may not remember it as BC-Predict).
   - 1. What was your overall experience of taking part in the study? If you can remember, tell me about the letter/leaflet you received?
     2. What did it tell you/what information was in it? How did it make you feel receiving risk information via letter/leaflet?
   1. What do you understand about what that risk means to you? Why do you think that?
   2. Can you tell me about whether you got in touch with the study team after you got your feedback?
   3. We have talked about how you felt when you received the result, how do you feel about the result now?

**Breast awareness**

1. How breast aware were you prior to receiving your below-average result?
   1. What does a below average risk result mean to you with regards breast awareness now (prompt: checking breast regularly). Why do you think that?

**Extended screening interval**

1. Research and healthcare professionals are suggesting that it could be safe to have screening less for women at below-average risk like you.
   1. What would you think about the possibility of going to your next screen at 6 years? (prompt: ask about length)
   2. What concerns do you think you or other women would have?
   3. What do you think are the positives to having less screens for below-average risk women?
   4. How would you feel about coming to screening less often? How would you have felt taking part in BC-Predict if less frequent screening had been a possibility?

**Extended screening interval – communication and role of HCPs**

1. How would you feel about being told you are now on a 6 year screening interval?
   1. Can you tell me about whether you would be reassured by this? Why? What about any concerns you would have?
   2. How would a HCP’s opinion influence how you feel about it? (prompt: explore different types of HCPs, e.g. doctor, mammographer) What would you want to know from the HCPs?
   3. Would you trust their advice?

**Extended interval – choice**

1. If it was a choice to come to screening every 6 years or stay at 3 years, how would you decide?
   1. What information would you need to know to make that decision?
      1. What would be reassuring to know?
   2. Would you make decision alone or discuss it with others? Why? (prompt: family, friends)
   3. What do you think are the main reasons why women at below-average risk would want to have screening less often?
   4. What do you think are the main reasons why women at below-average risk would want to stay at 3 years?

**Extended interval – information requirements**

1. Think about how you are/used to be invited to breast screening. If screening was to change for you, how would you like to receive information about coming to screening every 6 years?
   1. Leaflets, letters, face-to-face consultations?
   2. You found out your risk via letter. How appropriate would this have been if there had been a change to your screening?
   3. From whom would you like to receive information about going to screening every 6 years? (Prompts: BSP, GPs, Clinicians?)
   4. Who do you think this should be rolled out to for example, you are or have been in the breast screening programme already, other women will be invited for the first time

When should women receive information? (prompt: about the change)

1. Can you tell me what you know about the harms and benefits of screening? (prompt: this is not a test!)
   1. What do you understand about false positives/over diagnosis? {EXPLAIN HERE ABOUT FP/OD IF NEEDED}
   2. Does/Would knowing this information make you feel differently about having screening every 6 years?
2. Once a woman is on a longer interval, how do you think we could best support women like yourself who are at low risk if a change in screening was to be implemented?
   1. How do you think we could support women from different backgrounds? (prompt: if first language is not English or different educational backgrounds)
   2. What information would women at below-average risk need to remain breast aware if they were coming for screening every 6 rather than every 3 years?

**Extended interval – personal choice**

1. Would you personally want screening every 6 years instead of every 3 years?
   1. What would be your reason to have screening every 6 years (‘or not choosing’ every 6 years [dependent on answer])?

**Screening materials questions:**

1. Is there anything that needs to be in the letter/leaflet that isn’t?
2. As it is now would this letter/leaflet help you make a decision about coming to screening less frequently? Why? How would you feel about coming to screening every 6 years after reading this information?
3. We want to change these materials to include information about less frequent screening: is there anything that you think should be added to the leaflet to help you make a decision about coming to screening every 6 years? What changes do we need to make to the information you have just read?
4. What information should be in there about coming to screening every 6 years?
5. How else could we deliver this information to women?
6. Is there anything that you think shouldn’t be in there?
7. Is there anything else that you or other women would need? (prompt: contact details)

**Closing comments**

Thank you for talking to me today and sharing your views. Your insights will be invaluable when we come to pilot this change to screening for low risk women.

- Was there anything that you would like to add?
- Was there anything that you thought we would discuss and haven’t?
- Do you have any questions for me?
